# Examining the Eye Movement Behaviors Associated With Skilled Decision-Making by Elite Australian Rules Football Players

**DOI:** 10.3389/fspor.2022.899217

**Published:** 2022-07-08

**Authors:** Lael Kassem, Clare MacMahon, John Quinn, Sera Dogramaci, Bonnie Pang, Kylie A. Steel

**Affiliations:** ^1^School of Health Sciences, Western Sydney University, Sydney, NSW, Australia; ^2^Greater Western Sydney Giants Australian Football League, Sydney, NSW, Australia; ^3^Sport and Exercise Science, La Trobe University, Melbourne, VIC, Australia; ^4^Quinn Elite Sport Services, Sydney, NSW, Australia; ^5^Department of Health, University of Bath, Bath, United Kingdom; ^6^The MARCS Institute for Brain, Behaviour and Development, Sydney, NSW, Australia

**Keywords:** sports-cognition, decision making, eye-movement, sports-expertise, football

## Abstract

Exploration of eye-movement behaviors of humans can provide insight into the processes used to inform and make decisions, with a large body of research revealing general trends, especially in the sporting context. Despite this some questions remain within the sport context particularly for elite groups engaged in diverse sports, and the potential for this information to provide for training, development, and performance. Therefore, the purpose of this study was to examine the critical fixation points and durations associated with superior decision-making within an elite group of Australian Rules football players. To achieve this eye-movement behavior (fixations) and associated decision-making skills of (*N* = 27; Mage = 25.0 ± 3.7 yrs) elite Australian Rules (AR) football players were measured while they watched game-based video clips. The most skilled players made significantly faster decisions compared to less skilled players (*p* < 0.001), who also had significantly shorter total fixation duration (*p* < 0.0001). Further, analysis showed that the most skilled players spent more time fixating on potential options within an area of interest (*p* = 0.003). Thus, within a group of highly skilled group of athletes, distinctions can be made on perceptual-cognitive skills, for outcome decisions and decision processes. That is, skilled decision-makers appear to have more efficient visual search strategies, which may help them process visual information more effectively. Further, examination of these behaviors may aid sport science and coaching staff identify the process that can be refined to increase player ability between and within various teams.

## Introduction

The effective implementation of tactical skill in sport requires developed perceptual-cognitive and decision-making abilities, which tend to differ between skilled and less skilled athletes (Vaeyens et al., 2007; Dicks et al., 2019). Decision-making is the process of bringing about a required action which is informed by the perception and processing of relevant information. In the context of invasion sports, where scoring requires entering the opponent's territory, accurate and effective decision-making occurs when a player selects the best choice from a range of alternatives while managing high pressure constraints (Farrow and Raab, 2008; Bonney et al., 2020).

In dynamic situations that require tactical decisions, athletes must identify and attend to the most information-rich areas from the extensively distributed visual scene and make effective use of these areas (Williams and Williams, 1999). Perceptual-cognitive skills underpinning performance include a more efficient use of vision to scan the environment in order to extract relevant information (McRobert et al., 2011). Skilled athletes can generate accurate options of likely outcomes in any given situation based on the refined use of situational probabilities (McRobert et al., 2011). Researchers have uncovered unique gaze behaviors in skilled athletes that are qualitatively and quantitatively distinct from those of lesser skilled athletes. Skilled athletes appear to make better use of both their central and peripheral vision to attend to this information when making decisions (Mann et al., 2019). Despite these findings, some discrepancies still exist with the consistency of these findings within groups which often display a variation in ability.

In Australian Rules (AR) football, players are required to make fast and accurate decisions under both temporal and spatial constraints. Players need to interpret the actions of their opponents, their teammates and structured patterns of play in order to dispose of the ball and increase scoring affordances (Berry et al., 2008; Farrow and Raab, 2008; Larkin et al., 2020). Vaeyens et al. (2007) investigated eye movement behavior during decision-making scenarios in football and found that successful decision-makers relied on central search strategies that featured a greater number of gaze transitions between the player in possession of the ball and other meaningful areas of the display, reflecting a more structured search pattern and using the ball carrier as a constant frame of reference (Vaeyens et al., 2007; Mann et al., 2009, 2019). Specifically in AR football, high pressure decision-making contexts are characterized by scenarios where there are three or more disposal options (Parrington et al., 2013). These occur in ~26% of game play and thus, from an offensive perspective, the ability for a player to identify the correct disposal option is an important contributor to performance efficiency (Parrington et al., 2013). As such, researching the underlying mechanism of elite decision-making skill in order to establish quantifiable measures is an increasing interest within the sport science industry.

The ability to differentiate skilled tactical performers from their peers has value within established teams and the selection of new players in AR football. Currently, during talent identification practices, decision-making skill is assessed using relatively subjective assessments by coaches and recruiters who observe games and game footage (Larkin et al., 2020). This process has some limitations, as two recruiters might have differing perceptions of what constitutes good game play, thus the combination of both subjective and objective means of assessment can enhance player selection (MacMahon et al., 2019). For example, Woods et al. (2016) conducted a study using a multidimensional assessment approach that included physical (vertical jump, shuttle run), technical (handballing, kicking), and perceptual-cognitive (video-based decision-making task) variables. This more comprehensive approach demonstrated that more skilled players outperformed the less skilled players across each variable, with the video-based decision-making task being of particular interest due to the rarity of its use in player selection and particularly talent identification processes. The results showed that skilled players outperformed their less skilled peers at a rate of 92% correct decisions compared to 76%, respectively, thus presenting a possible means of assessing decision-making skill. The researchers, however, did not examine the mechanisms or processes underlying the final decision-making choice, specifically eye movement behavior and cognitive processes. These measures may provide even greater insights into the processes used in decision making relative to skill level.

Research exploring eye movement behavior is extensive and emphasizes working with stimuli specific to sport and athlete expertise, including game play and decision-making (Williams and Williams, 1999; Afonso et al., 2012). Many researchers have examined the mechanisms underlying decision-making skill by observing the visual search behaviors employed by participants as they make decisions for field-based sports in film-based tasks (Gabbett et al., 2008; Spittle et al., 2010; Larkin et al., 2011). The research has demonstrated that when making decisions, skilled athletes search the display in a more systematic and efficient manner than their less skilled counterparts (Mann et al., 2007, 2019). In many tasks, the first visible eye movement is to the location of some feature of importance in the task's execution (Land and Furneaux, 1997). Players then tend to fixate on this area while the visual system extracts information needed in the performance of the task. The time taken to process the information is a key discriminator between skilled and less skilled participants (Mann et al., 2019).

Skilled athletes have been shown to group perceptual information into larger and more meaningful units, enabling visual search based on their long-term working memory (LTWM) (Tenenbaum, 2003; Murphy et al., 2016; Roca et al., 2021). The LTWM of skilled athletes enables them to recognize the emergent features of a pattern of play early in its initiation, allowing for attention to be directed toward relevant areas and eliminate irrelevant information, thus requiring less processing time (Williams, 2000; Starkes and Ericsson, 2003). This facilitates anticipation and early decision-making (Ericsson and Chase, 1982; Mann et al., 2007, 2019). Additionally, perceptual-cognitive skill encompasses both visual search strategy and cognitive processes.

Within AR football, there is a paucity of research exploring eye movement behaviors specific to decision-making skill levels displayed by players across and within ability levels. A study by Lorains et al. (2013a), measured the visual search strategies of six AR football players during a 5-week speeded video decision-making training intervention. The study highlights the changes of visual search properties due to training and learning. The increase in average fixation duration in both training groups corroborates previous research that states experts have longer fixation durations (Helsen and Starkes, 1999). Furthermore, the study found that both the above real time and normal speed groups spent significantly more time fixating on the best option compared to the control group. These findings are in line with previous research, highlighting that elite decision-makers spend more time viewing areas of importance, and less time on irrelevant areas.

Building on findings from previous studies, the current study aimed to identify the key visual search strategies that underpin the perceptual-cognitive characteristics of decision-making skills of AR football players within an elite group. This was conducted using a video-based task, with eye movement recordings. We hoped to gain further understanding of the role that eye-movements play in the decision-making process which may then be used to establish a method to discriminate this skill within an elite football cohort. We hypothesized that superior decision-makers would demonstrate more efficient visual behaviors involving longer average fixation durations on potential best options within the display.

## Materials and Methods

### Participants

Twenty seven male elite AR football players (*M*_*age*_= 24.9 ± 3.77 yrs) from a professional metropolitan football club volunteered to participate in the current study. Player experience at a professional level ranged from first year drafted players (0 games played) to 15 years (282 games played), (*M*_*games*_= 72.4 ± 75.2) with each player specializing in specific positions (midfielders = 10, forwards = 9, and defenders = 8). All participants provided informed consent prior to involvement with the study in accordance with the ethics protocol for this study which was approved by the University Human Research Ethics Committee (H12131).

### Video Footage

The video footage for this study was extracted from stored game-day footage captured during the 2017 season which was provided by the professional AR football club involved in this study. Two different viewing perspectives were available: behind the goals and broadcast (televised footage). The inclusion criteria for the selected videos were based on the protocol used by Woods et al. (2016) and Larkin et al. (2020), where the researchers determined the inclusion criteria of video clips for the study which required three main features: (1) An offensive perspective (the team was in possession of the ball at the decision-making moment); (2) Player in possession of the ball had three to five teammates available to kick or handball to control for the number of possible decisions participants had: and (3) A lead time of ~15 s prior to the critical decision-making moment.

### Decision-Making Options

A total of 20 videos were initially identified by the lead researcher for the task which were then reviewed the remaining research team members similar to Larkin et al. (2020), and finally by three highly experienced coaches, each with a minimum of 10 years coaching at state or higher level (Lorains et al., 2013b). Each coach was asked to identify the best passing options for the player in possession of the ball. Coaches independently ranked their top three options on a 3-2-1 scale, with three being the most “ideal” option and one being the “least ideal” option. Only videos where all three coaches' agreed upon the three options were included, resulting in a total of 14 videos used for the decision-making task of the study.

### Eye-Tracking Technology

**Figure 1:**
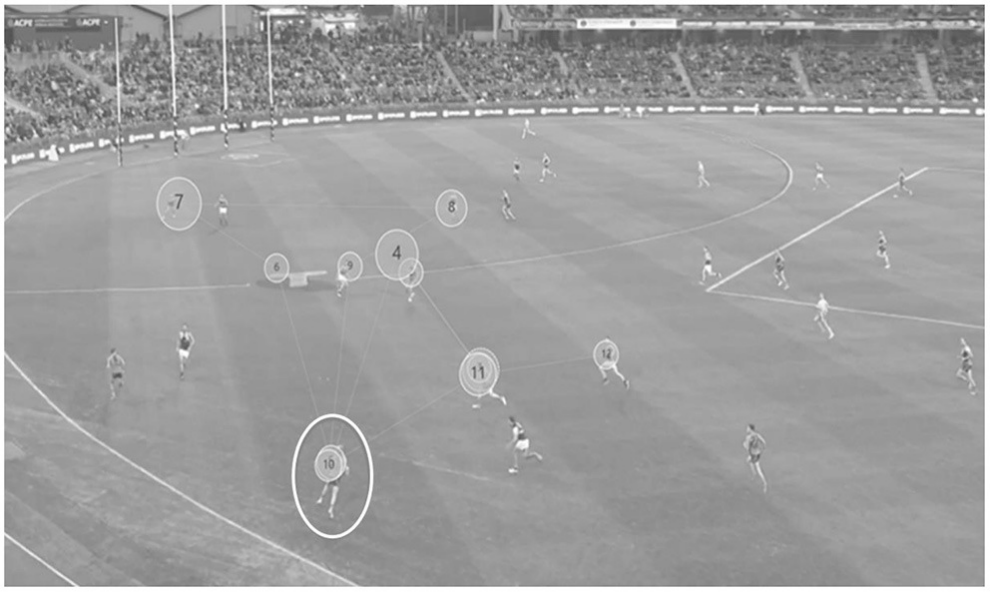
Example Tobii Pro lab data output indicating visual search patterns. The total number of fixations to a part of the display is indicated in the circle, by chronological order. The size of size of the circles signifies the fixation duration with larger circles indicating a longer duration. The player circled in white is in possession of the ball, and the team in gray uniforms are on offense.

Tobii Pro Glasses 2 (Tobii AB, Stockholm, Sweden) eye-tracking glasses were used to measure the eye movement behavior of the participants, while taking part in the video-based decision-making task. The glasses collected visual search data at a rate of 50 Hz with 82° horizontal and 52° vertical visual angle of precision. During the decision-making task several variables were measured as outlined in Table 1. Figure 1 provides an example visual representation of the eye movement recording output obtained. In addition, the visual display was divided in to four areas for further analysis into participants' gaze behaviors: Defenders (D), Options (O), Player with the ball (B), Space (S). Options was defined as areas within the display, where the ball could be passed to by the player in possession of the ball. This included the three options agreed upon by the panel of coaches, in addition to other options where unanimous agreement was not attained. For example, a suitable teammate or the goals as two separate options. Note, not all teammates were classified as options.

**Table 1:** Eye movement behavior variables.

**Variable**	**Description**
Fixation	The periods of time where the eyes are relatively still, holding the central foveal vision in place so that the visual system can take in detailed information. Tobii I-VT (attention) filter classifier was set at 100°·s^−1^.
Fixation duration	The length of time of each individual fixation(s).
Total fixation duration	The sum of all fixation durations.

### Procedures

All testing was conducted in one lab location with each participant fitted with the eye-tracking glasses, followed by individual calibration on arrival. This calibration process required the participant to stand in front of the projected image on a screen, with their eyes focused on a red dot in the center of the screen. The software completed the calibration process automatically within a 30 s time frame. Participants remained standing in this position for the duration (10–12 min) of their data collection session to elicit a greater stimulus response compatibility effect, as previous research has shown that responses are faster and more accurate when a spatial stimulus array matches a spatial response array (compatible mapping) than when it does not match (incompatible mapping) (Roca et al., 2014). The height and distance measurements were adapted to each participant, ensuring eyesight was in the center of the display and allowing for greatest compatibility.

**Figure 2:**
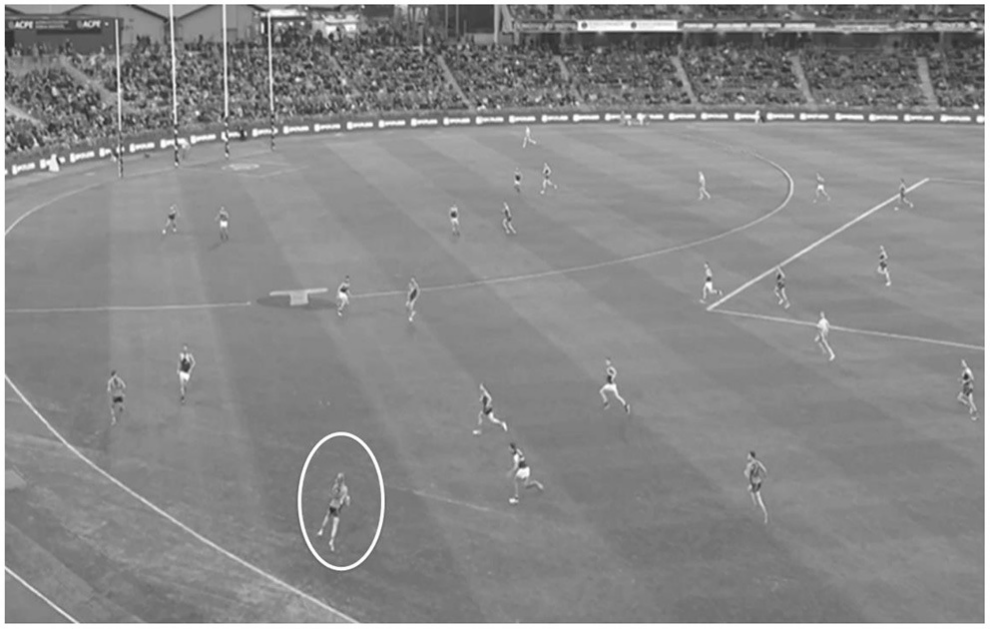
An example of a critical decision-making moment, used for the film-based task. For this example, the player with the ball is circled for reference, with the team in the gray uniforms on offense.

Participants were given instructions on the standard procedure which consisted of two familiarization clips, followed by 14 test clips, played in a random order. Each clip paused at the critical offensive decision-making moment, at which point participants were to imagine they were the player in possession of the ball and decide what the next best move would be, at which point they provided a verbal indication of their decision. Participants' score was matched with the scale (3-2-1) agreed upon by the three independent coaches. Participants received a 0 if they selected an option outside of the coaches' opinions or if they did not make decisions within the allocated 4 s. This time pressure was used to reflect the temporal constraints of real game scenarios (Woods, 2015). An example of the final critical moment of a video clip is shown in Figure 2.

### Statistical Analysis

All results were collated, and various statistical procedures implemented using Statistical Package for Social Sciences (SPSS, Version 22). Descriptive statistics for all variables were calculated and are reported as mean ± standard deviation (SD). An alpha level of *p* < 0.05 was selected as the criterion for significance for all statistical procedures. A mixed model binomial logistic regression was used to account for the multiple responses, allowing the variance between videos to predict whether cases can be correctly classified (skilled vs. less skilled) from the independent variables.

Response accuracy (RA) was used to classify participants into groups. We categorized responses into two groups to allow for comparison and to predict the probability of observations falling into the skilled group (3 = Skilled; 2, 1, 0 = Less Skilled). This was applied to each individual video; statistical analysis was performed based on each specific video and participants response category to each video separately. To examine the potential ability to use eye movement data to predict skill level, a Receiver Operator Characteristic (ROC) curve was used to determine the test's ability to predict skilled decision-makers. All recordings were coded within the Tobii Pro Analyzer software, which produced data for all the following independent variables: (a) Decision Time (DT): measured from the moment the video paused at the critical decision-making moment until the participant made a verbal sound (seconds); (b) Duration of fixation(s): the duration of fixation (across all locations) measured in seconds; and (c) Total duration: the sum of all individual fixation durations.

Two researchers independently coded the data *via* Tobii Pro Analyzer, which involved manual mapping of fixation points and allocation of the different areas of interest. Once coding was complete, the data were checked for consistency and agreement in coding locations and categories. The level of agreement between coders was very high (>95%). Any differences in coding were clarified through group discussions.

## Results

### Decision Time

**Figure 3:**
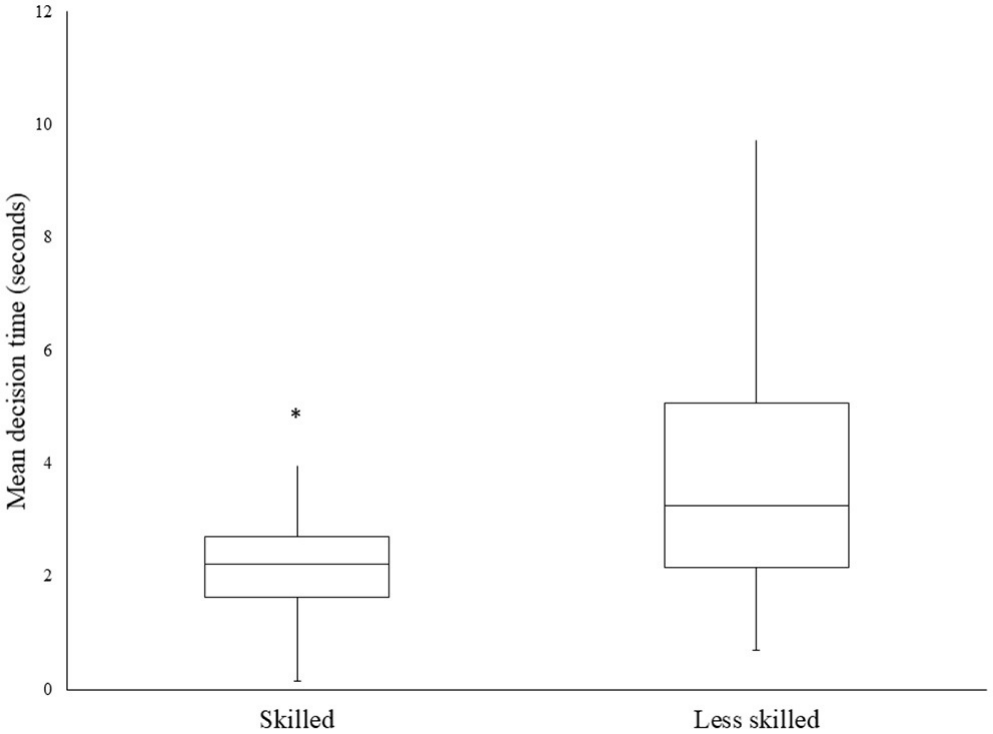
Mean decision time between “skilled” and “less-skilled” participants. *Significantly different from less skilled *p* < 0.0001 (95% CI [0.302, 0.507]).

On average, the skilled group made significantly faster decisions (2.21 ± 0.76 vs. 3.71 ± 1.92) as seen in Figure 3. A binomial logistical regression was performed to ascertain the effects on DT for the likelihood that the participants scored a 3 (most skilled) in the video-based decision-making task. For every 1 s increase in DT, there was a 0.391 95% CI [0.302, 0.507] reduced chance of being in the skilled group, *p* < 0.0001.

### Eye Movement Behavior

A binomial logistic regression was performed to highlight the probability of being skilled based on the fixation duration. Several significant differences in visual search patterns were observed between skilled (*n* = 15) and less skilled (*n* = 11) participants as shown in Table 2.

**Table 2:** Mean visual search characteristics between groups.

**Variable**	**Skilled Mean ±SD**	**Less skilled Mean ±SD**	**Odds ratio [95% CI]**	***P*** **-value**
Fixation duration (s)	2.21 ± 0.84	2.75 ± 0.93	0.496 [0.36, 0.684]	0.0001
Number of fixations (*n*)	4.59 ± 2.20	5.99 ± 2.84	0.783 [0.702, 0.874]	0.0001

*Odds ratio indicated the likelihood of being in the “less skilled group.” For example, for every increase in fixation, a player increases their chance of being in the less skilled group by 0.783*.

### Fixations Across Different Locations

**Figure 4:**
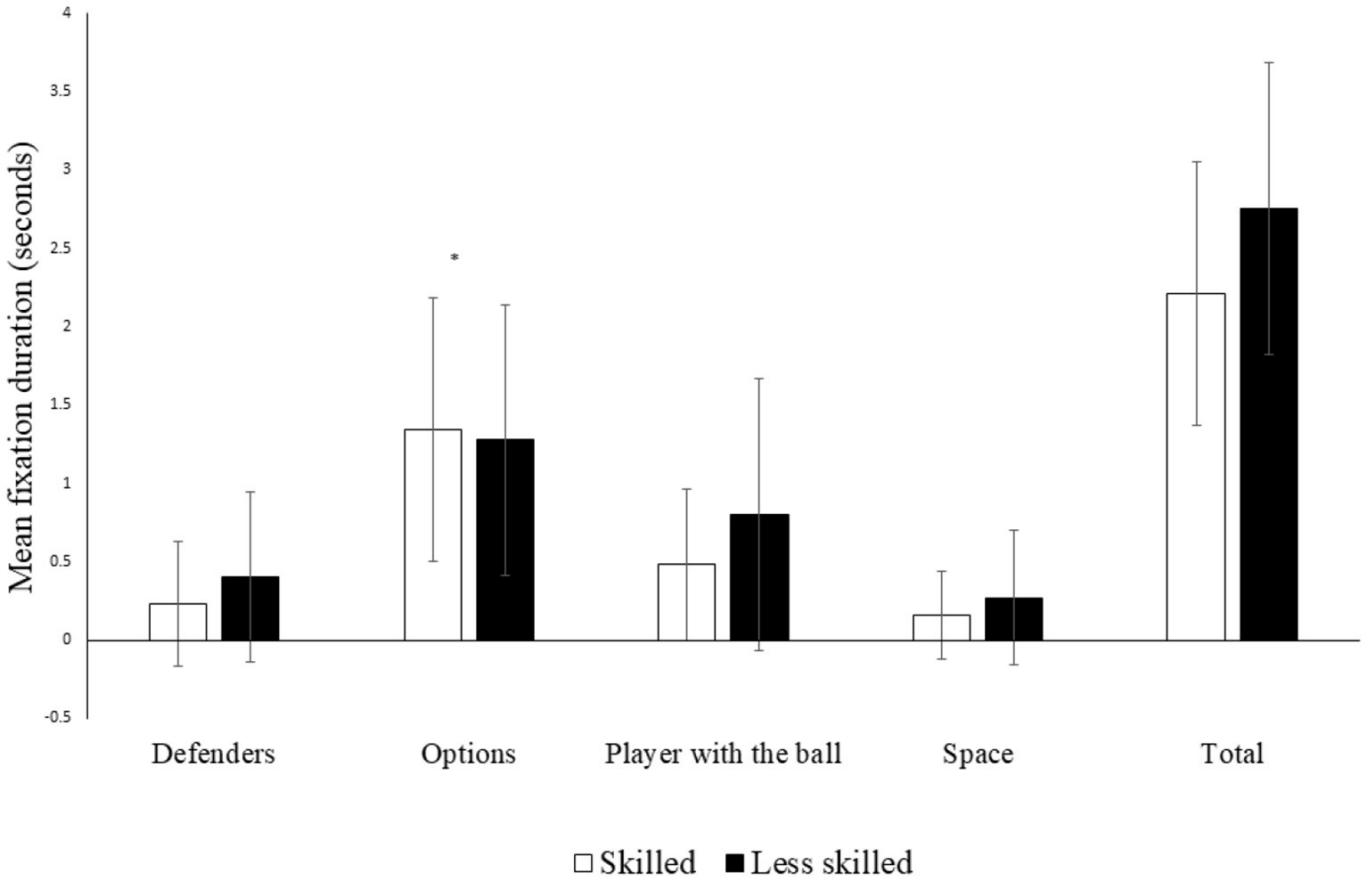
Mean duration of fixations across different areas, between skilled and less skilled groups. *Significantly different *p* = 0.003.

The skilled decision-makers demonstrated longer fixation durations on their teammates (1.34 ± 0.84 s vs. 1.28 ± 0.86 s) compared to any other areas in the display, which was a significant predictor for making skilled decisions *p* = 0.003. The odds ratio indicated that for every 1 second increase fixating options, players improved the chance of being in the skilled group by 0.587 95% CI [0.411, 0.838] (Figure 4).

## Discussion

There is limited understanding of more objective measures of skilled tactical performance, specifically decision-making, for elite level AR football players. Current procedures and practices remain a largely subjective process where coaches and recruiters observe footage and pass judgment on a player's decision-making ability, which presents a major limitation to broader testing protocols aimed at development and training. The current study aimed to quantify key characteristics of perceptual-cognitive and decision-making skills, namely the eye movement behavior of elite AR football players. To achieve this, participants wore eye-tracking glasses to capture their visual search strategies while taking part in a film-based decision-making task. The data provides additional information that allows further understanding of the underlying mechanisms for elite athlete decision-making which can provide a rationale for more objective means of assessment.

A within-task criterion was used to stratify participants into skilled and less skilled groups based upon their decisions made and matched against an expert panel of coach ratings. The results supported the hypothesis that skilled participants made significantly faster decisions (*p* = 0.0001) compared to their less skilled counterparts. These findings support previous research which suggests that skilled players have a faster processing time, where they can differentiate between areas of importance without scanning the whole scene (Mann et al., 2019). It also supports the concept of long-term memory (Ericsson and Delaney, 1999), which illustrates that experts can retrieve information from memory faster, and therefore the information processing is more efficient with each situation encountered, and as experience accumulates. This can be observed through reduced fixation duration and shorter decision times (Gegenfurtner et al., 2011). Findings also align with the Take the First heuristic, which states that skilled players pick the first best decision that comes to mind, and the longer they scan other options, the worse their performance becomes (Johnson and Raab, 2003).

Further, the skilled group spent significantly more time fixating on teammates/options within the display compared to the less-skilled group (*p* = 0.003). Similar results were found by Vaeyens et al. (2007) when investigating eye movement behaviors of youth soccer players. Results indicated that the skilled group fixated on the player with the ball for longer periods of time, and that players fixate on a central point and use peripheral vision to monitor movements around this point (Dicks et al., 2019). A critical point of difference between Vaeyens et al. (2007) and this study however is the sport in question (Soccer vs. AR football). Soccer is played on a rectangular pitch with 11 on field players as opposed to AR football which is played on an oval pitch with 18 on field players. As such key differences within the dynamic display due to differing patterns of movement, available space, and number of players, provide varied reasons for the difference in the central fixation point (Teammates/options vs. player with the ball). However, this does not change the overall visual search strategy used for making skilled decisions, that being the use of a peripheral vision while fixating on a central key point in the display. The findings support the *Information reduction theory* (Gegenfurtner et al., 2011), which proposes that experts selectively allocate attentional resources by devoting greater attention to task relevant information. Consequently, experts tend to utilize the time gained through efficient attentional strategies for better decision-making process.

The current study highlights that perceptual-cognitive and decision-making skill can be captured *via* more objective testing designs that use video stimuli. These designs allow the control of several variables and comparisons between individuals, especially for decision-making. By also combining video-based methods with eye-tracking technology, more data from a wider range of variables can be captured and analyzed, providing a deeper understanding of the decision-making process in fast ball sports.

Lorains et al. (2013b) used a film-based decision-making training intervention using above real time video clips vs. normal speed video clips, over an 8-week period. At the post test the above real time training group showed the greatest overall improvement, followed by the normal speed group, with no notable improvements for the control group who did not receive training. The above real time training group also maintained some improvements at the first retention test 2 weeks after training ceased, which was not the case for the normal speed group. All groups approached pre-test decision-making levels at the second retention test in week 19 which was 11 weeks with no training. The trends present in this study provide a baseline for the effectiveness of video-based methods when testing decision-making abilities in AR football that can be enhanced by the inclusion of eye movement behavior assessment.

Future studies should extend upon the current findings by considering the individualized nature of decision-making to account for player position and perceived ability. For example, the coach's indication of ‘best option’ may not be congruent with the players' knowledge of what is possible given their abilities and skills. Examining decision-making and eye-movement behavior would also benefit from exploration in an *in-situ* context, though technological design and cost remain prohibitive at this time.

## Conclusion

The skills needed to achieve excellence in a sport are complex and multifaceted, highlighting the importance of a comprehensive and objective skill identification process which assesses all determinants of game play. The current study was able to demonstrate that by using a video-based decision-making task paired with eye-tracking technology decision-making can be objectively assessed to distinguish the skill levels more accurately between players at a professional AR football club. This can then be used to develop and enhance decision-making skill in elite/professional athletes, increasing the validity and reliability of player selection processes within AR football, with the potential to cross over or be utilized in other sports (such as soccer, rugby league, rugby union, field hockey).

## Data Availability Statement

The raw data supporting the conclusions of this article will be made available by the authors, without undue reservation.

## Ethics Statement

The studies involving human participants were reviewed and approved by Western Sydney University Human Research Ethics Committee. The patients/participants provided their written informed consent to participate in this study.

## Author Contributions

LK, KS, and SD contributed to all aspects of this study. CM contributed significantly to the data analysis and manuscript development at each stage. BP and JQ were co-supervisors on this project and contributed to participant recruitment and manuscript development at each stage. All authors contributed to the article and approved the submitted version.

## Funding

This study did not receive any funding beyond that of a Ph.D. scholarship for the LK from Western Sydney University, Greater Western Sydney Australian Rules football club and Skins™ compression clothing.

## Conflict of Interest

The authors declare that the research was conducted in the absence of any commercial or financial relationships that could be construed as a potential conflict of interest.

## Publisher's Note

All claims expressed in this article are solely those of the authors and do not necessarily represent those of their affiliated organizations, or those of the publisher, the editors and the reviewers. Any product that may be evaluated in this article, or claim that may be made by its manufacturer, is not guaranteed or endorsed by the publisher.

## References

[B1] Afonso J. Garganta J. McRobert A. Williams A. M. Mesquita I. (2012). The perceptual cognitive processes underpinning skilled performance in volleyball: evidence from eye movements and verbal reports of thinking involving an in situ representative task. J. Sports Sci. Med. 11, 339–345. 24149208 PMC3737875

[B2] Berry J. Abernethy B. Côté J. (2008). The contribution of structured activity and deliberate play to the development of expert perceptual and decision-making skill. J. Sport Exerc. Psychol. 30, 685–708. 10.1123/jsep.30.6.685 19164836

[B3] Bonney N. Berry J. Ball K. Larkin P. (2020). The development of a field-based kicking assessment to evaluate Australian Football kicking proficiency. Res. Q. Exerc. Sport. 91, 73–82. 10.1080/02701367.2019.1647331 31502925

[B4] Dicks M. Araújo D. van der Kamp J. (2019). Perception-action for the study of anticipation and decision-making, in Anticipation and Decision-Making in Sport, eds Williams M. Jackson R. (Abingdon, VA: Routledge), 181–199.

[B5] Ericsson K. A. Chase W. G. (1982). Exceptional memory: extraordinary feats of memory can be matched or surpassed by people with average memories that have been improved by training. Am. Sci. 70, 607–615. 7181217

[B6] Ericsson K. A. Delaney P. F. (1999). Long-term working memory as an alternative to capacity models of working memory in everyday skilled performance, in Models of Working Memory: Mechanisms of Active Maintenance and Executive Control, eds Miyake A. Shah P. (Cambridge: Cambridge University Press), 257–297. 10.1017/CBO9781139174909.011

[B7] Farrow D. Raab M. (2008). A recipe for expert decision-making. Dev. Sport Expertise 137–159. 10.4324/9780203934937

[B8] Gabbett T. J. Carius J. Mulvey M. (2008). Does improved decision-making ability reduce the physiological demands of game-based activities in field sport athletes? J. Strength Condition. Res. 22, 2027–2035. 10.1519/JSC.0b013e3181887f34 18978606

[B9] Gegenfurtner A. Lehtinen E. Säljö R. (2011). Expertise differences in the comprehension of visualizations: a meta-analysis of eye-tracking research in professional domains. Educ. Psychol. Rev. 23, 523–552. 10.1007/s10648-011-9174-7

[B10] Helsen W. Starkes J. (1999). A multidimensional approach to skilled perception and performance in sport. Appl. Cogn. Psychol. 13, 1–27. 10.1002/(SICI)1099-0720(199902)13:1<1::AID-ACP540>3.0.CO;2-T

[B11] Johnson J. G. Raab M. (2003). Take the first: option-generation and resulting choices. Organ. Behav. Hum. Decis. Process. 91, 215–229. 10.1016/S0749-5978(03)00027-X

[B12] Land M. F. Furneaux S. (1997). The knowledge base of the oculomotor system. Philos. Trans. R. Soc. London Ser. B Biol. Sci. 352, 1231–1239. 10.1098/rstb.1997.0105 9304689 PMC1692006

[B13] Larkin P. Berry J. Dawson B. Lay B. (2011). Perceptual and decision-making skills of Australian football umpires. Int. J. Perform. Anal. Sport 11, 427–437. 10.1080/24748668.2011.11868562

[B14] Larkin P. Marchant D. Syder A. Farrow D. (2020). An eye for talent: the recruiters' role in the Australian Football talent pathway. PLoS ONE 15, e0241307. 10.1371/journal.pone.0241307 33137113 PMC7605670

[B15] Lorains M. Ball K. MacMahon C. (2013a). Expertise differences in a video decision-making task: Speed influences on performance. Psychol. Sport Exerc. 14, 293–297. 10.1016/j.psychsport.2012.11.004

[B16] Lorains M. Ball K. MacMahon C. (2013b). An above real time training intervention for sport decision-making. Psychol. Sport Exerc. 14, 670–674. 10.1016/j.psychsport.2013.05.005

[B17] MacMahon C. Bailey A. Croser M. Weissensteiner J. (2019). Exploring the skill of recruiting in the Australian Football League. Int. J. Sports Sci. Coach. 14, 72–81. 10.1177/1747954118809775

[B18] Mann D. Williams A. M. Ward P. Janelle C. M. (2007). Perceptual-cognitive expertise in sport: a meta-analysis. J. Sport Exerc. Psychol. 29, 457. 10.1123/jsep.29.4.457 17968048

[B19] Mann D. L. Causer J. Nakamoto H. Runswick O. R. (2019). Visual search behaviors in expert perceptual judgements, in Anticipation and decision making in sport, eds Williams A. Jackson R. (Abingdon, VA: Routledge), 59–78.

[B20] Mann D. L. Farrow D. Shuttleworth R. Hopwood M. (2009). The influence of viewing perspective on decision-making and visual search behaviour in an invasive sport. Int. J. Sport Psychol. 40, 546–564.

[B21] McRobert A. P. Ward P. Eccles D. W. Williams A. M. (2011). The effect of manipulating context? specific information on perceptual-cognitive processes during a simulated anticipation task. Br. J. Psychol. 102, 519–534. 10.1111/j.2044-8295.2010.02013.x 21752003

[B22] Murphy C. P. Jackson R. C. Cooke K. Roca A. Benguigui N. Williams A. M. (2016). Contextual information and perceptual-cognitive expertise in a dynamic, temporally-constrained task. J. Exp. Psychol. Appl. 22, 455. 10.1037/xap0000094 27936856

[B23] Parrington L. Ball K. MacMahon C. (2013). Game-based analysis of handballing in Australian Football. Int. J. Perform. Anal. Sport 13, 759–772. 10.1080/24748668.2013.11868687

[B24] Roca A. Ford P. R. Memmert D. (2021). Perceptual-cognitive processes underlying creative expert performance in soccer. Psychol. Res. 85, 1146–55. 10.1007/s00426-020-01320-5 32200407

[B25] Roca A. Williams A. M. Ford P. R. (2014). Capturing and testing perceptual-cognitive expertise: a comparison of stationary and movement response methods. Behav. Res. Methods 46, 173–177. 10.3758/s13428-013-0359-5 23794270

[B26] Spittle M. Kremer P. Hamilton J. (2010). The effect of screen size on video-based perceptual decision-making tasks in sport. Int. J. Sport Exerc. Psychol. 8, 360–372. 10.1080/1612197X.2010.9671958

[B27] Starkes J. L. Ericsson K. A. (2003). Expert performance in sports: advances in research on sport expertise. Human Kinetics. 10.5040/9781492596257

[B28] Tenenbaum G. (2003). Expert Athletes: An Integrated Approach to Decision Making. Expert performance in sports. p. 191–218.

[B29] Vaeyens R. Lenoir M. Williams A. M. Philippaerts R. M. (2007). Mechanisms underpinning successful decision-making in skilled youth soccer players: an analysis of visual search behaviors. J. Mot. Behav. 39, 395–408. 10.3200/JMBR.39.5.395-408 17827116

[B30] Williams A. M. (2000). Perceptual skill in soccer: implications for talent identification and development. J. Sports Sci. 18, 737–750. 10.1080/02640410050120113 11043899

[B31] Williams D. K. Williams J. G. P. (1999). Visual Perception and Action in Sport. London: E & FN Spon.

[B32] Woods C. T. (2015). The development of an objective multi-dimensional approach to talent identification in junior Australian football (Doctorate Thesis). Edith Cowan University, Joondalup, WA, Australia. Available online at: http://ro.ecu.edu.au/theses/1672

[B33] Woods C. T. Raynor A. J. Bruce L. McDonald Z. Robertson S. (2016). The application of a multi-dimensional assessment approach to talent identification in Australian football. J. Sports Sci. 34, 1340–1345. 10.1080/02640414.2016.1142668 26862858

